# Use of Forceps

**Published:** 1892-04

**Authors:** Edward Cranch

**Affiliations:** Erie, Pa.; Bureau of Obstetrics, I. H. A.


### USE OF FORCEPS.

Edward Cranch, M. D., Erie, Pa.

(Bureau of Obstetrics, I. H. A.)

The above title does not have the definite article, because it is not proposed to give an exhaustive treatise on the subject, but, accepting the standard indications for the use of forceps, such as impaction, unusual delay, certain malpositions, and so forth, as given in modern text-books, it is now proposed to give a few details of indication for the use of forceps that have presented themselves to the writer in his twenty years of practice.

A frequently-met condition is this: labor progressing fairly, in best vertex position, but waiting in the excavation, without apparent cause. First, be sure that the position of the head is normal, a very important point; then, if delay shows only increasing elongation of the head, with no apparent progress in at least an hour, then, applying the forceps, which is easily done in the excavation, one or other of two conditions will be found to have been most often the retarding cause: wrapping of the cord about the neck, or the presence of a hand alongside the face. This last condition, though frequently met with, does not appear to have been attended to by writers.

Mention was made of the importance of locating the exact position of the head. In early practice it was thought enough to know that the head was presenting, to make it proper to apply the forceps, if delay occurred, without careful exploration, resulting in frequent scarring of the face in transverse positions, laceration of ears, and the mother's perineum, if the vertex was posterior, and fruitless efforts at extraction, with subsequent disastrous resort to version, in cases with the hand preceding the head, or in face presentations, in most of their positions.

Now it is deemed best, on the slightest doubt in locating the head, to wait long enough to be sure there is delay ; then proceed, with anæsthetics, if necessary, to locate the head, finding the parietal protuberances or the ear, and tipping or rotating the body till the head presents properly, or proceeding at once to perform version, if the head does not come to time.

One of the most useful expedients, whose original author is not just now remembered, is the tilting of the head by one, or, better, two fingers on the crown, well toward the mother's back, when the vertex is forward, but flexion being delayed, the regular descent has not taken place.

The writer has been called in consultation and performed this little sleight of hand to the great surprise of the attendant, and the satisfactorily speedy delivery of the mother. The manœuvre sometimes has to be repeated at the inferior strait, with good results. In general, it is the writer's belief that the use of forceps should not be too long delayed, nor should they be used through the final exit, if the labor-pains are abundantly strong, though here is a nice field for the exercise of judgment in individual cases.

We should not be too afraid of laceration of the perineum, and a preliminary incision may guide the laceration when it appears inevitable.

The appearance of laceration is not always an evidence of the previous use of forceps, as some professors have asserted, nor is it always an evidence of their ignorant or clumsy use, as others have urged. Much doubtless depends on the shape of forceps employed. They should not be too thick, nor the shanks too widely separated ; on the other hand, they must not be slender enough to bend and slip off, nor narrow enough to exercise injurious compression.

Much erroneous opinion is extant as to the speed that should attend delivery with forceps ; it cannot be fixed, but must vary with circumstances, as must the amount of force to be used.

A thorough knowledge of and due reflection on the anatomy of the parts, and their ascertained degree of rigidity, will prevent recklessness and insure success where success is possible.

In abnormal positions of the head, especially if low down, it is often better that the forceps should be applied, even with some risk, than that the graver risk of version should be undertaken.

It is a good rule never to go to a case without the forceps, one or two pairs, in hand, or in easy reach, for thereby much annoyance and risk will be averted.

